# Targeting the “Sweet Side” of Tumor with Glycan-Binding Molecules Conjugated-Nanoparticles: Implications in Cancer Therapy and Diagnosis

**DOI:** 10.3390/nano11020289

**Published:** 2021-01-22

**Authors:** Nora Bloise, Mohammad Okkeh, Elisa Restivo, Cristina Della Pina, Livia Visai

**Affiliations:** 1 Department of Molecular Medicine, Center for Health Technologies (CHT), INSTM UdR of Pavia, University of Pavia, Viale Taramelli, 3/B-27100 Pavia, Italy; mohammadkhalil.okkeh01@universitadipavia.it (M.O.); elisa.restivo01@universitadipavia.it (E.R.); livia.visai@unipv.it (L.V.); 2 Medicina Clinica-Specialistica, UOR5 Laboratorio Di Nanotecnologie, ICS Maugeri, IRCCS, Pavia, Via Boezio, 28-27100 Pavia, Italy; 3 Dipartimento di Chimica, Università Degli Studi di Milano e CNR-ISTM, Via C. Golgi, 19, 20133 Milan, Italy; cristina.dellapina@unimi.it

**Keywords:** aberrant glycosylation, glycan-binding molecules, lectin, antibody, tumor targeting and therapy, nanomedicine

## Abstract

Nanotechnology is in the spotlight of therapeutic innovation, with numerous advantages for tumor visualization and eradication. The end goal of the therapeutic use of nanoparticles, however, remains distant due to the limitations of nanoparticles to target cancer tissue. The functionalization of nanosystem surfaces with biological ligands is a major strategy for directing the actions of nanomaterials specifically to tumor cells. Cancer formation and metastasis are accompanied by profound alterations in protein glycosylation. Hence, the detection and targeting of aberrant glycans are of great value in cancer diagnosis and therapy. In this review, we provide a brief update on recent progress targeting aberrant glycosylation by functionalizing nanoparticles with glycan-binding molecules (with a special focus on lectins and anti-glycan antibodies) to improve the efficacy of nanoparticles in cancer targeting, diagnosis, and therapy and outline the challenges and limitations in implementing this approach. We envision that the combination of nanotechnological strategies and cancer-associated glycan targeting could remodel the field of cancer diagnosis and therapy, including immunotherapy.

## 1. Introduction

Although significant progress towards the development of anticancer drugs, science still faces many problems concerning the side effects of chemotherapy. Various nanostructures-based drug delivery systems have been synthesized to improve the therapeutic selectivity of this treatment [1,2,3]. Nonetheless, despite the rapid growth of nanotechnology in medicine, the promises of cancer therapy based on effective targeting and drug delivery is still a challenging task. The efficiency, safety, and selectivity of this therapy can be improved by surface conjugation of nanoparticles with molecules able to actively target cancer cells and cellular uptake while minimizing their immunogenicity [4]. Current efforts are devoted to developing methods that exploit cancer features for selective nanoparticle targeting. Herein, we will provide a brief review of the current status of nanotechnology-based strategies by using glycan-binding molecules to identify aberrant glycosylation patterns: an approach which, in turn, would enhance the specificity of cancer-targeting, diagnosis, and therapy. 

## 2. Targeting Nanoparticles to Cancer

Passive targeting, which exploits the enhanced permeability and retention (EPR) effect, is the most extensively studied approach for nanoparticle delivery to cancer tissue [5]. Despite the great potentiality of this strategy, its effectiveness is reduced by the heterogeneity of the EPR effect and the physiological barriers related to it. It is also clear that the physicochemical properties of the nanomedicine (including size, shape, and surface chemistry), severely affect the nanoparticles uptake at the tumor site [6,7]. It is well accepted that the optimization of nanoparticle (NP) design and an active targeting mechanism can overcome these limitations, giving rise to a more specific accumulation of nanoparticles in tumors rather than undesired localization. Typically, active targeting involves the functionalization of the surfaces of the nanoparticles with one or more targeting moieties, with the capacity to recognize specific receptors or antigens that are either uniquely expressed or upregulated on the tumor cells relative to healthy tissues: a concept which has triggered the development of numerous methods for structural modification of NPs.

Active targeting, however, is also not without limitations. First, ligand-nanoparticles can be rapidly cleared from the circulation by the reticuloendothelial system (RES) or can accumulate in unwanted organs, such as the spleen and liver. Second, the heterogeneous nature of the tumor and the adaptability of cancers are barriers to reach an absolute active targeting. The selection of an appropriate targeting ligand is critical for optimizing the efficiency of active targeting and is mainly governed by the specific characteristics of the target tumors, such as the target receptor and its expression [8,9]. Currently, a variety of targeting ligands has been tested to deliver the drug in both in vitro and in vivo models. These include nucleic acids, nucleic acid-based aptamers, small molecules, synthetic peptides, protein domains, sugars, and antibodies [7,10]. As reviewed by Friedman et al., each ligand class possesses unique properties and can be conjugated to the NP structures by different strategies of conjugation strategies, including physical adsorption and chemical conjugation [11]. The properties of targeting ligands and their successful conjugation with nanoparticles significantly impacts the application of NPs in targeted imaging, diagnosis, and cancer therapy. Thus, in the choice of NP ligands, the researchers should also consider different aspects, including the specificity for the antigen, the ease of conjugation, the stability, and the cost of fabrication: factors which will ultimately impact the biological interactions of NPs, such as cellular uptake and circulation time. Antibodies (Abs), for example, are smart ligands with high specificity and diversity of targets, but with a high cost for fabrication and conjugation. 

On the contrary, small chemical molecules (such as folate, anisamide, phenylboronic acid) are of small size and have very low costs, but generally low specificity as their targets are also expressed in normal tissues [9]. NPs surface functionalization offers great potential for targeting and integration of therapeutic agents [12], a point which explains the remarkable efforts that have been made to improve NPs surface features to increase their interaction with cancer cells. We also note the development of novel, bioinspired, approaches to surface coating NPs which exploit the versatility and multifunctionality of plant polyphenols. Several groups have reported promising results using tannic acid (TA), one of the most well-known plant-derived polyphenols, to functionalize the NPs surfaces [13,14,15,16]. TA was chosen as a functional ligand because of its well-known biocompatibility, biodegradability, and the bulky nature of this hydrophilic, which allows for effective water exchange on the hydrophobic surfaces of NPs [17]. From a biological point of view, TA is particularly promising in those cancer types that overexpress the epidermal growth factor receptor (EGFR), as TA regulates its activation and downstream signaling pathways, eliciting the apoptosis process. Aguilera produced multifunctional tannic acid nanoparticles with an extremely high entrapment efficiency of the active principle and targeted to EGFR, which were only toxic for the cancer cells [18]. Moreover, TA was successfully employed to coating Ultrasmall iron oxide nanoparticles (USIONPs), with uptake by cancer cells being demonstrated by in vitro experiments [13,14]. Another ligand, also of interest, that showed the potential to be used as an active tumor-targeting ligand is quinic acid (QA). QA—a synthetic mimic of sialyl-Lewis x (SLe^x^), and its derivates, interacting with selectin receptors expressed on tumor cells and tumor endothelium—has been shown to promote the targeting and the internalization of NPs and as a result, is proving to be valuable tumor-targeting ligand [13,19]. Overall, we can state that both the production and identification of new and selective ligands have never been greater and the discovery of new cancer biomarkers is particularly valuable for improving the efficiency of cancer treatment.

## 3. Aberrant Glycosylation in Cancer

Extensive research has revealed the critical role of glycans alteration in cancer biology. Glycans, simple or complex carbohydrates, are present in different classes of glycoconjugates (i.e., glycoproteins, proteoglycans, and glycolipids) on the outer layer of the cell surface, forming the so-called glycocalyx [20]. The glycocalyx is involved in almost all fundamental functions across all multicellular organisms, acting as structural and modulatory player of numerous physiological processes, such as cell–cell communication, cell adhesion, proliferation, differentiation, and immune response regulation [21]. Glycosylation is a highly regulated and most frequent process in which a saccharide moiety is attached to a protein by the expression and activity of specific enzymes [20]. The huge array of glycan function it is mainly related to the vast heterogeneity of glycans, itself dependent on different factors: the nature of monosaccharide subunits, the various combination in which they can be linked, the anomeric configuration of linkage (α or β), the branching degree, and the further modifications of terminal structures (i.e., sulfation, phosphorylation, and acetylation). 

**Figure 1:**
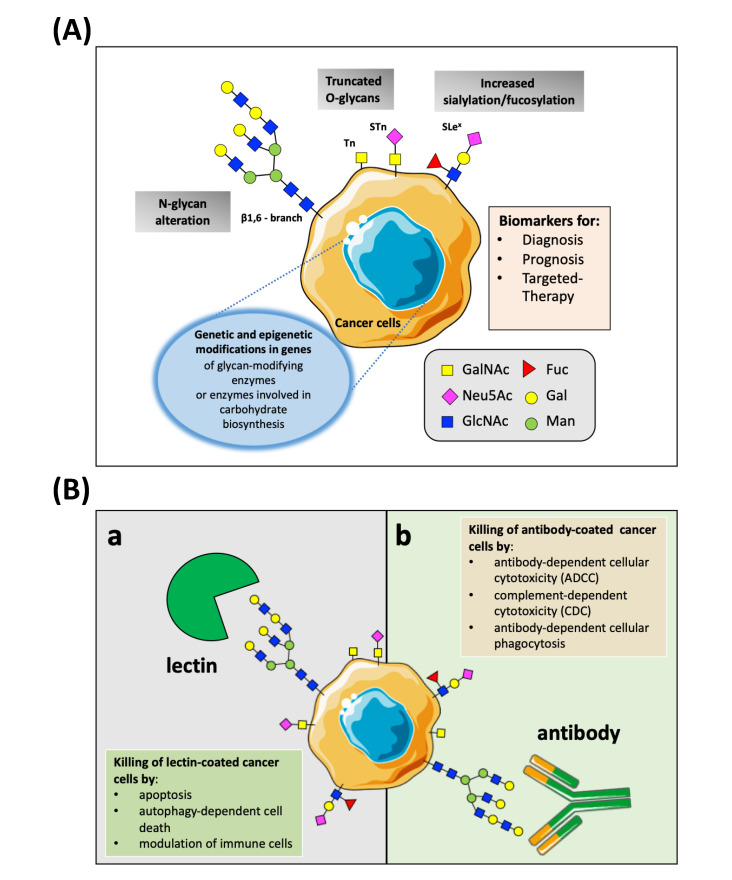
(**A**)**.** Schematic representation of the characteristic pattern of cancer-associated glycosylation. The three main glycan changes (i.e., altered branching of N-glycans, the expression of truncated O-glycans, and increased sialylation/fucosylation) can be found in cancer due to genetic or epigenetic alterations in genes of glycosylation enzymes-glycosyltransferases and glycosidases. Abbreviations: GalNAc, N-acetylgalactosamine; Neu5Ac, sialic acid; GlcNac, N-acetylglucosamine; Fuc, fucose; Gal, galactose; Man, mannose; Tn, Tn antigen; STn, sialyl-Tn antigen; SLe^x^, sialyl-Lewis^X^. (**B**). Overview on the anti-tumor mechanisms of natural lectins and anti-glycan antibodies that can be advantageous for the cancer nanomedicine. (**a**) The binding of lectins to tumor-associated carbohydrates antigens present on the surface of cancer cells elicits cell killing through apoptosis, autophagy, and immunomodulatory activity against cancer. (**b**) Abs can recognize TACAs and induced cell death via different mechanisms, such as antibody-dependent cellular cytotoxicity, complement-dependent cytotoxicity, and antibody-dependent cellular phagocytosis.

Typically, based on the binding site of the glycan to a protein, there are two types of glycosylation: N-linked or O-linked glycosylation. In the first ones, the glycans are attached to the amide group of asparagine side chains, whereas, in the second type, the glycans are added to the hydroxyl oxygen of serine and threonine side chains [20,22]. Aberrant glycosylation has been shown to frequently occur in oncogenic transformation and progression in all types of human cancers. The common cancer-glycan alterations, classified as tumor-associated carbohydrates antigens (TACAs), include truncated O-glycans (Tn-antigen, sialyl-Tn antigen, and Thomsen-Friedenreich antigen (TF), branched N-glycans (β1,6-branching N-acetylglucosamine), aberrant fucosylated and sialylated glycans (known as tumor sialoglycans) [23,24] (Figure 1A). Abnormal glycosylation in cancer cells can be linked to a different mechanism, such as under/overexpression of glycosyltransferases and glycosidases; changes in the general reorganization of glycosyltransferase topology, alterations of glycosyltransferase localization within the secretory pathway, and, lastly, the availability or abundance of sugar nucleotide donors. These possibilities are not mutually exclusive and one or other may become predominant in a variety of situations. Genetic and epigenetic alteration modifies the expression of these glycan-modifying proteins and varies among tumor types. Numerous studies have demonstrated that the altered glycans are active players throughout cancer development and progression by significantly altering the protein landscape, originating unique cancer fingerprints at the cell-surface [23].

For these reasons, in the last years, the tumor-associated carbohydrate antigens have been largely used as prognostic biomarkers in the clinical diagnosis of patients with neoplastic diseases [25,26,27]. Furthermore, several findings suggest that this utility could be expanded also to that of therapeutic targets, providing an unequivocal label for tumor cell recognition and therefore improving the specificity of tumor treatments [27,28,29,30], with enormous implications for the monitoring of conditions and for personalized cancer therapy. Recently, Thomas et al. have discussed in a review the fundamental role of glycans in regulating tumorigenesis and tumor progression and provided insights into the influence of glycans in the current tactics of targeted therapies with particular reference to glycoconjugate drugs, glycan-based vaccines, glycosylation specific inhibitors/mimetic, and targeted nanotherapies [28]. The identification of cancer-specific biomarkers has oriented the field of nanomedicine toward the use of glycans for developing new feasible cancer nanomedicine strategy.

## 4. Decorating Nanoparticles Surfaces with TACAs-Binding Molecules

Modifications in glycosylation machinery can generate dramatic alterations in the antigenic profile at the tumor cell surface. Though glycosylation is complex and challenging, the research on glycosylation is evolving and therefore many groups are exploring the use of cancer-associated glycans for potential clinical application, such as cancer targeting and therapy.

In this area, the glycan-binding molecules (GBPs) can be used to discriminate between tumor and normal cells. Some GBPs, such as the lectins and anti-glycan Abs, can also trigger cancer cell death due to their anti-tumor properties (Figure 1B). These capabilities make GBPs attractive options as tumor-targeting ligands that can be used to increase the selectivity and efficiency of nanoparticles towards cancer cells and enhance their concentration in the tumor sites. The following discussion highlights some recent and prominent results of NPs-based strategies to tackle TACAs by decorating NP’s surfaces with natural lectins and anti-glycan Abs and illustrates the implications of these developments for targeting, diagnosis, and therapy of cancer disease. We also describe, in the same way, the new promising active targeting strategies with the potential to target glycosylation aberrations.

### 4.1. NPs Conjugated with Natural Lectins: A Well-Trod Path

#### 4.1.1. Lectins

Lectins are proteins present in all branches of the evolution tree since they have been found in all organisms, from bacteria to fungi and from plants to animals [31]. They have been found to act as glycan-specific ligands, specifically binding one or more specific structural epitope per cancer-related glycoproteins. In particular, lectins recognize the glycans mostly via a network of hydrogen bonds between the ring oxygen atom and multiple hydroxyl groups of the carbohydrate residues and oxygen atoms, amide and hydroxyl groups of the protein, as well as electrostatic interaction [32]. These glycan-binding molecules have very specific affinities towards for only one or two types of monosaccharides whose recognition is crucially dependent on how the saccharide is presented by the glycoprotein. In the cancer field, plant lectins have attracted great interest in cancer studies for cancer diagnosis, imaging, and treatment thanks to their ability to identify aberrant glycans expressed by neoplastic cells [33,34,35,36]. Recently, Bhutia et al. documented the anticancer and immunomodulatory activities of the major plant lectins that have been studied in this field (i.e., the legume lectins, ribosome-inactivating proteins type-II lectins, *Galanthus nivalis* agglutinin (GNA) lectins, chitin-binding lectins, and jacalin), illustrating also their potential mechanism of action in cancer cells [33]. On the contrary, a complete description of both animal and plant lectins was provided by Yau et al. [37]. In general, the anticancer activity of lectins is primarily mediated through apoptosis in various cancers by different mechanisms of action (Figure 1B(a)). For example, it was reported that plant lectins interact with sugar-binding receptors present on the plasma membrane and, after their internalization through endocytosis altered ROS generation, targeted to mitochondria to generate ROS and release cytochrome c into the cytoplasm, thereby activating p21-Foxo1a-Bim-mediated apoptosis and extrinsic apoptotic pathways [33]. Several lectins have also been demonstrated to successfully exert antitumor effects through the implication of autophagy [38], or the inhibition of cancer cell migration [39]. Furthermore, lectins have also been reported to act as potent immune stimulators, which may contribute to the elimination of cancer cells from the human body [40]. Although lectins have enormous potential in the cancer field, and some of them have exhibited preclinical and clinical significance [34], to date, only Mistletoe lectins (MLs) have been extensively studied in clinical trials to assess their anticancer potentials [41]. It remains, however, that, while showing these promising binding specificities, lectins, unfortunately, tend to have weak binding affinity stability, poor selectivity for individual sugars, and difficulties in production and purification and toxicity concerns toward human health. All of which limits their exploitation both in practical assays application and clinical translation [32,42]. Currently, lectins are routinely used in academic laboratories for carbohydrate structure characterization, for purification of glycoproteins, and for labeling of specific epitopes expressed on the cell membrane.

#### 4.1.2. Lectins-NPs

In the context of cancer diagnostics and therapeutics, several pre-clinical studies have explored the functionalization of lectins onto multifunctional NPs surfaces, with exciting up-front results. Table 1 summarized some of the recent lectin-based nanotechnology strategies. For instance, recently, Martínez-Carmona et al. used the plant Concanavalin A (ConA) in an innovative nanodevice based on doxorubicin (DOX)-loaded MSNs where different building blocks were assembled for targeted bone cancer treatment [43]. In this system, the presence of lectin grafted to a polymeric shell significantly enhanced the selectivity towards cancer cells whilst preserving the healthy bone cells. In another work, it was shown that the Wheat germ agglutinin (WGA) decoration on anti-tumoral drugs-loaded nanoparticles considerably improved nanoparticles cellular uptake and exhibited synergistic cytotoxicity [44]. Devices for the early in situ detection of cancer and their precursors prior to their malignancy transformation are clearly desirable. In this field, Chen et al. reported their promising findings using lectins for the functionalization of fluorescent mesoporous silica nanoparticles (MSNs) as an endoscopic contrast agent for in situ diagnostic imaging of premalignant colonic lesions [45], demonstrating the targeting role of these molecules. Significantly, the theragnostic (simultaneous diagnosis and treatment) application of lectins is nowadays gaining relevance in cancer nanotechnology. For example, lectin-conjugated NPs can be used for the early detection of cancer cells, this makes NPs attractive for use in cancer diagnosis, and at the same supports the release of drugs exclusively into the tumor site. To achieve this goal, Chowdhury et al. successfully fabricated nanocomposites based on graphene quantum dots, ConA, and Fe_3_O_4_ to explore their multifunctional applications in electrochemical cancer cell detection and DOX-controlled delivery [46]. These researchers showed that these nanocomposites, when deposited on platinum electrodes, were able to detect cancerous HeLa cells over normal endothelial cells clearly showing that ConA effectively-recognized and attached to the glycans’ environment of cancer cells. In addition, they also concluded that the specificity towards cancerous cells achieved by the ConA attachment made the nanocarriers as promising nanoplatform for drug release triggered by external magnetic fields.

### 4.2. NPs Conjugated with TACAs-Binding Antibodies: An Explored Road

#### 4.2.1. Anti-TACAs Abs

The anti-tumor-associated carbohydrates Abs are also widely studied as glycan-targeted ligands [52]. They typically have much lower affinities than antibodies for recognizing proteins or peptide antigens (by 3–5 orders of magnitude). Additionally, the glycosylation complexity of biological samples restricts the use of the anti-glycan antibodies to immunohistochemical staining, Western blotting, and ELISA assays for discovering, detecting, and purifying carbohydrates linked with a broad range of cancer tissues both in basic research and clinical applications [53]. On their molecular mechanism, the occurrence of the same glycan epitope on a wide range of glycoproteins and glycolipid can hugely increase their biological activity. However, like other Abs, the binding of anti-glycan Abs to cancer cells can elicit antibody-dependent cellular cytotoxicity (ADCC), complement-dependent cytotoxicity (CDC), and phagocytosis, activating in all these processes numerous receptor-mediated signaling pathways, resulting in cell apoptosis and oncosis (Figure 1B(b)). [54,55]. As recently reviewed by Mantuano and colleagues, several monoclonal antibodies (mAbs) exist to target aberrant glycans, which are in clinical use or pre-clinical studies, and are known to stimulate ADCC and/or CDC mechanisms [56]. For example, Dinutuximab is a chimeric mAb generated against disialoganglioside GD2 and approved for the treatment of high-risk neuroblastoma pediatric patients [57]. Almost 95% of neuroblastomas consistently express the GD2 antigen, while only low levels are detected on healthy tissues [58]. GD2 can be considered a tumor-associated antigen and well-suited as a target for cancer therapy. Several other mAbs have been designed to target Lewis antigens (Le), expressed by a wide variety of tumor cells. Examples of such mAbs are Hu3S193 and BR96 targeting the Lewis Y glycan antigen and used in clinical trials for treating lung, breast, and colorectal carcinoma [59,60,61]. Nonetheless, the use of mAbs to reveal and detect glycans has some limitations. Indeed, despite the high spectrum of antiglycan mAbs—refers to the Database for Anti-Glycan Reagents (DAGR) containing more than 1000 unique anti-glycan monoclonal antibody entries (https://ccr2.cancer.gov/resources/Cbl/Tools/Antibody/)—there are quite a few antiglycan antibodies which are commercially available [53]. The development of highly selective and specific monoclonal antibodies to glycans/glycoproteins, then, remains a challenge and represents an obstacle for the progress of the glycoscience field [62]. The major hurdles are related to both the intrinsically poor glycan immunogenicity and similarities in carbohydrate sequences. Additionally, the isolation of pure and structurally defined carbohydrates for antibody generation and characterization can be extremely difficult and laborious. It was shown that glycan density, flexibility, and polymer backbone rigidity can influence the functional affinity (avidity) of glycan-binding protein [63,64]. Thus, many efforts are being made to enhancing glycan-binding antibodies affinity, which is typical with an equilibrium dissociation constant (K_D_) in the micromolar range, by exploiting the recent advancement in glycome findings, protein engineering and phage displace technologies for producing mAbs holding two or multiples glycan-binding sites and with superior affinity [32]. 

#### 4.2.2. Anti-TACAs mAbs-NPs

Among the biological ligands, Abs, or immunoglobulins (Ig), the large glycoproteins which are found in all vertebrate life forms, and antibody fragments, represent major nanoparticles targeting and therapeutic tools to fight cancer due to their specific binding to receptors overexpressed on cancer cells. Very promising results have also been obtained conjugating monoclonal, chimeric, and humanized antibodies with a variety of nanoparticles, which hold great promise to enhance therapeutic efficacy and circumvent severe side effects [65]. Despite the great potentiality of antibodies, it remains the case that inadequate pharmacokinetics, poor tissue accessibility, and therapy resistance represent barriers for the therapeutic antibodies and limit their clinical application, suggesting the need for novel delivery strategies [66]. The conjugation with nanoparticles to improve the delivery of the antibody has achieved significant outcomes both in vitro and in clinical studies [11,67]. Several strategies have been developed and exploited to immobilize antibodies on the surface of NPs while preserving antigen-binding ability and yielding stable antibody-conjugated NPs. Nevertheless, antibody targeting of nanoparticles is still challenging, with many concerns such as the antigen-binding affinity, that must remain high after the conjugation, the specificity of the conjugation, the stability of the Ab-NPs conjugated during the circulation time, and importantly the immunogenicity of the conjugated [11,68]. While the targeting of nanoparticles with anti-TACAs Abs may undoubtedly achieve significant results in tumor identification and therapy, facing the aforementioned challenges related to the anti-glycan antibodies is imperative for their application in cancer nanomedicine. It is probable that, due to all these difficulties, nanoparticle functionalization with anti-glycan Abs will require further exploration. To our knowledge, the first experimental attempts to link anti-glycan antibodies to nanoparticle platforms have involved the anti-GD2 Abs or their fraction in the targeting and treatment of neuroblastoma [69,70]. As recently summarized by the Rodríguez-Nogales group, anti-GD2 Abs were efficiently conjugated with metallic, lipidic, and polymeric nanoparticles for tumor-specific targeting of neuroblastoma, with high potential to serve as a multifunctional therapeutic nanoplatform [69]. Indeed, theragnostic NPs open a new dimension in tumor-targeted therapeutic strategies that combine in simultaneous diagnosis and treatment of a disease at a curable stage. In particular, Baiu et al. provided the first line of evidence that iron nanoparticles conjugated with the clinically relevant antibody hu14.18K322A (humanized monoclonal anti-GD2) were able to target human neuroblastoma with high levels of specificity and at levels detectable by magnetic resonance in a murine flank xenograft model, suggesting their application as theragnostic nanoplatform for other GD2+ malignancies [71]. Recently, another group improved the delivery of SN-38 (a topoisomerase-I inhibitor) by using an antibody against GD2 to target SN-38 loaded NPs into human tumors [72]. They showed that this delivery system was antigen-specific in vitro and in vivo, the tumor penetration by SN-38 was drastically higher in mice receiving the targeted nano-drug delivery system than non-targeted NPs or the free drug, establishing a proof of concept that GD2 targeting of NPs using mAbs could have clinical potential to enhance anti-tumor activity while reducing toxicity effects. These promising approaches based on anti-GD2 Ab-NPs suggest that they may well be applied in the near future to target and treat GD2-expressing tumors. Disialoganglioside GD2 is indeed highly expressed not exclusively by almost all neuroblastomas, but also by most melanomas and retinoblastomas, and by to a more variable degree, by many osteosarcomas and soft tissue sarcomas [73]. Heavily glycosylated proteins, such as proteoglycans, also form an interesting array of targets for tumor imaging in addition to tumor-associated glycans. Heparan sulfate proteoglycan, such as Glypican-1 (GPC-1), has been reported as a potential pancreatic cancer (PC) biomarker [74] as its expression is greater in human PC than normal cells [75]. Huang et al. established a new multifunctional nanoprobe designated for detecting pancreatic cancer cells (PC) [76]. The author showed that the conjugation with GPC-1 antibody enabled the functional imaging probe to effectively target GPC-1-expressing PC cells both in vitro and in vivo, suggesting the application of this newly targeted-system for detecting PC cells in the clinic. Table 2 summarized some of the recent antibody-based nanotechnology strategies.

### 4.3. Other Anti-Glycans Approaches to Target NPs towards Cancer

Although analyzing and determining the aberrantly glycosylated motifs is, therefore, an important diagnostic and therapeutic goal, it is clear that new glycan-specific receptor alternatives are highly needed due to the limited availability and challenges of lectins and glycan-specific antibody molecules. In this context, various novel and interesting models and solutions have opened a new avenue for the recognition of TACAs on cancer surfaces (Table 3). For example, current advances in this area have focused on aptamers (Apt) to target carbohydrates with charged moieties, such as the sialic acids [82,83]. Apt are a class of short nucleic acid (DNA or RNA), that are preferable to anti-glycan mAbs due to their affordable production cost, negligible immunogenicity, and small size [9,32]. Mucin1 (MUC1) is a glycoprotein known as a tumor-associated antigen that is aberrantly overexpressed in several cancer cells [84]. Consequently, anti-MUC1 Apt can be used for targeted drug delivery to cancer cells [85,86]. Recently, Jafari et al. used the anti-MUC1 Apt to target chitosan NPs containing Docetaxel and insulin-like growth factor receptor 1(IGF-1R) Silencer siRNA (gene silencing by small interfering RNA) to SKBR3 breast cancer cells [87]. This demonstrates that this novel targeted co-delivery system could enhance the cellular uptake of NPs and profoundly decreased the pathways involved in tumorigenesis and metastasis. Another effective way to target glycans is through peptides. Indeed, the numerous advantages of peptides (such as low cost of production, good stability, and ease of conjugation to the surface of NPs at a high density due to their small size), make them appropriate anti-glycans ligands to target cancer cells. Different examples can be found in the literature [9,88,89]. For instance, Rossez et al. prepared ultrasmall particles of iron oxide (USPIOs) conjugated with disulfide constrained heptapeptide, that were identified using a screening phage display, for the early detection by magnetic resonance imaging (MRI) of colon cancer using human gastric mucin MUC5AC as a specific marker [90]. More recently, Zhao et al. obtained successful outcomes using dextran-coated iron oxide NPs conjugated to the near-infrared fluorescent dye Cy5.5 and to a uMUC1-specific peptide (EPPT) as a probe for MRI and fluorescence optical imaging [89]. Another promising strategy involves the use of small molecules, such as boronic acids, for the construction of synthetic receptors for glycan recognition. Boronic acids covalently bind 1,2- and 1,3-diol groups found in carbohydrates [32]. In particular, diboronic acid compounds have been found to recognize cell surface cancer-associated glycans in situ, such as SLe^x^ with high levels of specificity [91]. There is great potential for the use of boronic aid and derivates application for the development of targeted nanoparticles towards cancer cells for drug delivery, cell imaging, and therapy. Soy protein NPs bearing phenylboronic acid (PBA) on their outer surface were used to improve the tumor microenvironment (TME) targeting and penetration of NPs inside the tumor [92]. It was also observed that the incorporation of the phenylboronic acid group into chitosan NPs imparted a surface charge-reversible characteristic to the NPs, increasing the internalization of NPs into 2D and 3D cell models [93]. Another noteworthy approach includes the use of synthetic carbohydrate receptors created through Molecularly Imprinted Polymers (MIP) as a general toolbox for the specific recognition of cancer cells [94,95]. In the MIP technique, a polymer network is obtained around a template molecule (i.e., a glycoprotein expressed on the cell surface) in order to generate artificial binding sites which, upon removal of the template, can be occupied by their target [96]. Very recently, monosaccharide-imprinted fluorescent NPs were used for targeting and imaging of hepatoma carcinoma cells (HepG2) and mammary cancer cells (MCF-7), suggesting a possible additional application also for the synthesis of monosaccharide-imprinted plasmonic NPs for targeted photothermal therapy [95]. In addition, other innovative and appealing methods and strategies to focus on the glyco-specific issue, also in combination with NPs, have been developed. For instance, several polysaccharides such as chitosan, chondroitin sulfate, alginate, hyaluronic acid (HA) have been used to improve the active targeting of nanoparticle drug delivery systems [30,97,98,99]. Concerning HA, many studies have shown that HA-based nanoparticles can serve to efficiently target the CD44 glycoprotein, whose isoforms and their altered glycosylation are correlated with metastatic properties of human cancer [100,101,102]. Lastly, interesting models have been proposed and applied to various glycosylation targets, especially in combination with nanoparticle carrier systems. The interaction of endogenous GBPs with aberrant glycan structures significantly influence many pathological events, such as tumor progression. In this context, lots of new carbohydrate-modified NPs have been proposed to target the GBPs expressed on the immune or non-immune cells or in the extracellular space [103,104,105].

## 5. Possible Implications in Cancer Nano-Immunotherapy

Nanoparticles provide us with unique opportunities to improve the safety and the efficacy of cancer immunotherapy, a treatment that aims to support the re-awakening of the immune system to attack aberrant cancer cells [107]. Increasing evidence has pinpointed TME as the major target of nanomedicine. NPs have the potential to interact with the TME in unexpected ways and to ultimately and critically affect performance and tumor response [108,109]. A wide range of NPs with different physicochemical characteristics has been developed to stimulate the immune system and battle cancer [66,110]. NPs have been developed to activate and target dendritic cells, to deliver cytokines, genes, and antibodies, and also to target immunosuppressive cells in the TME, offering a promising strategy to eliminate this tumor-induced immunosuppression and stimulate the antitumor effect of immune cells [111]. The tumor microenvironment is indeed formed, besides the cancer cells, of many types of infiltrating immune and stromal cells interacting in a complex environment of soluble and matrix molecules. These interactions are critical in determining the fate of the tumor [112]. 

Notably, changes in glycosylation—such as β1,6-branched N-glycans, overexpression of tumor-associated mucins, and altered surface sialylation—are found not only in tumor cells but also in cancer associated-stromal, vascular and immune cells [113,114], which points toward a central role of glycobiology in immune responses. These changes are indeed involved in tumor development by hiding or uncovering specific ligands for endogenous lectins, including C-type lectin, Siglec, and galectin families that are typically widely expressed on immune cell surfaces, such as dendritic cells, neutrophils, and tumor-associated macrophages (TAMs) macrophages [115,116,117,118,119]. TAMs are key components of the leukocytic infiltrate in tumors and are highly plastic cells mostly due to their capability to adjust their metabolism and reprogram their phenotype into proinflammatory M1 (anti-tumor) or anti-inflammatory M2 (pro-tumor) phenotype to respond to changes in TME [120]. As recently discussed by Mantuano et al. [113], growing data suggest the important role of glycosylation in regulating tumor-associated macrophage polarization and differentiation. It has been observed that most growth factor receptors and molecules expressed on the surfaces of macrophages are N-glycosylated and that these modifications influence their interaction with endogenous lectins, determining the polarization of TAMs [121,122,123]. For example, the N-glycan containing terminal β-galactose can be recognized by galectins, a class of endogenous lectin that bind specially to β-galactoside sugars, modulating the macrophages functions [123]. Given this, several attempts are underway to develop therapies to foster TAMs polarization into cancer-suppressive phenotypes, such as sugar agonists or antagonist treatments and nanoparticles-based approaches [109,124]. 

A recent review has shed light on the use of CD24 as a target for cancer therapy [125]. CD24 is a highly glycosylated protein which is primarily expressed by immune cells but is often overexpressed in human tumors. In cancer, by interaction with endogenous Siglec-10 on TAMs, CD24 protects cancer cells from phagocytosis by acting as a phagocytic inhibitor (a“do not eat me” signal). CD24 has a great therapeutic potential and has become the target for different therapy formats. For instance, unconjugated mAbs were used to blockade the CD24–Siglec-10 interaction with the aim of strongly increasing the phagocytosis of all CD24-expressing human tumors tested. As this glycosylated protein may have additional ligands [126], we can suppose it may well be itself the target of other molecules, such as anti-glycan molecules, also conjugated with NPs, to prevent phagocytic inhibition via Siglec-10. In parallel, new insights have been obtained on the immunotoxicity of plant lectins toward cancer cells and their role in the reinforcement and modulation of the innate (anti-cancer) immunity (i.e., mononuclear phagocyte system). It was found that Jacalin, a noncytotoxic plant lectin extracted from the seeds of *Artocarpus integrifolia,* can recognize the macrophage surface, inducing the level of polarization toward the antitumor phenotype [127]. Similarly, Abrus lectins derived peptides were able to stimulate TAMs to reduce the expression on mannose receptors, to stimulate nitric oxide (NO) production and IL-1 secretion, and to increase phagocytosis [128].

Although the alterations in glycome occurring in TME are still poorly understood, these stimulating findings highlight the key role of protein-carbohydrates interaction in the TME crosstalk, a point which may inspire the development not only of glyconanoparticles able to mimic the carbohydrates on target cells to block these biorecognition systems, but also to produce anti-glycan nanoparticles to boost the anti-tumor activities of immune cells or to prevent their interaction with cancer cells. With this in mind, we envisage that the development of cutting-edge technologies (in mass spectrometry-based methods) for the glycoconjugates characterization, together with the progress in the nanoparticles production, gene expression analyses, or editing (CRISPR, clustered regularly interspaced short palindromic repeats-Cas9 gene editing), it may help us to gain new knowledge of this emerging field and contribute to our ability to uncover the function of glycosylation changes in glycan structures on the cell surface of cancer and immune cells. Advanced analytical techniques have enabled us to explore and pave the way for the future use of glycan-binding molecules-nanoparticles as immunomodulatory agents to re-educate TAMs and immune cells to fighting cancer cells.

## 6. Potentialities and Obstacles of Anti-Glycan NPs in Cancer Research

As mentioned above, several researchers have recently described various strategies for cancer “sweet side” targeting, through the design of nanoparticles bearing anti-glycan molecules, such as lectins or antibodies. Moreover, the appealing features of these functionalized nanoparticles enable them to be used in a variety of cancer areas including drug delivery systems. The advantages of lectin and antibodies, which include better targeting and intrinsic anti-tumor activities, promote the usage of these molecules in the structure of NPs to increase the selectivity of nanoparticles towards cancer cells. Even with some success in animal models, this new strategy has limitations that must be extensively considered before allowing them to be utilized in vivo. Therefore, the development of anti-TACAs NPs needs rigorous consideration. The pros and cons of the target, the tumor-associated glycans, are important. TACAs properties offer the following major advantages [88]: (i) their dense epitope expression on a wide range of tumors, but not in healthy tissue, resulting in a denser accumulation of anti-glycan molecules; (ii) their expression on the outermost layer of cell surface layer, making them easily accessible by targeting moieties; (iii) their ability to be expressed in target multiple tumor-associated proteins, then providing a more efficient targeting than single protein targeting. 

Nevertheless, it is true that the complexity of glycosylation remains a challenge; the fact that glycans are not very immunogenic is a major disadvantage [129]. Second, the researchers should consider the features of glycan-binding molecules. Lectins and glycan-binding mAbs have been identified as powerful tools to target NPs towards TACAs expressed on tumor cells. Nevertheless, as we described in the previous sections of this review, these molecules have different advantages and disadvantages. Lectins display relatively weak affinities for monosaccharides, with dissociation constants (K_D_) in the millimolar range, and poor selectivity for individual sugars explained by the absence of deeper binding pockets. The multiple interactions with terminal sugars due to their homo-oligomeric structures allow for superior affinity and selectivity. However, concerns related to their purification, immunogenicity, and toxicity must be tackled prior to their application as targeting ligand [31,32]. Similarly, there remain several obstacles with mAbs, despite their high specificity, such as large size, which can severely decrease the accessibility to the tumor, toxicity, and high cost of manufacturing, hindering their translation into clinic use [9,53,130]. On the other hand, the TACAs binding affinity, and the intrinsic anti-tumor activities of both lectins and antibodies, make them attractive and efficient ligands for the active targeting of NPs to the tumor. Some of the limitations may be partially lifted by using diverse and interesting approaches targeting cancer-associated glycans, such as aptamers, but more research is required to understand its safety and efficacy prior to clinical application. Third, an important consideration concerns the NPs themselves. The numerous advantages of NPs—including delivery control, increased payload stability, and solubility, enhanced permeability, and retention, magnetic and optical properties—make them suitable vehicles to deliver a wide spectrum of molecular cargos [1,131]. However, physicochemical properties (i.e., size, shape, rigidity, or surface) profoundly influence the large-scale distribution of NPs and the ligand conjugation [132,133,134,135,136]. 

In a recent paper, we demonstrated that increasing the gold nanoparticles size can improve the stability and the conjugation efficiency of Trastuzumab improved. In addition, we observed that the antibody retained its folding and anticancer activity after conjugation with NPs [10]. Consequentially, researchers should critically evaluate the effects of the NPs properties while at the same time not overestimating the effect of biological ligands. We finished by highlighting that it is worth exploring an effective “stealth“ approach to reach a better biodistribution, enhanced recognition, and effective internalization into the cancer cell [137]. Different biodegradable polymers were tested for coating NPs while minimizing immunogenicity reactions [138]. Nowadays, PEG coating is widespread but has some drawbacks in low drug loading efficiency if the drug is conjugated to the PEG chains [139]. Recent research has led to the development of biocompatible nanomaterials, especially for the controlled release of therapeutic molecules (chemotherapy, siRNA, small molecules of different natures, etc.) to cancer cells. For instance, Fernandes et al. developed PLGA-based glycoengineered NPs for selective delivery of chemotherapy (5-FU and paclitaxel) to SLeA-expressing gastric cells, with minimal off-target affinity for healthy tissues [97]. Furthermore, a winning approach for controlled drug release and its prevention from enzymatic degradation is represented by the NPs surface functionalization using polysaccharides. Polysaccharides display helpful abilities, such as increasing the solubility of low soluble or insoluble drugs, enhancing the blood circulation time, maximizing therapeutic efficacy, and minimizing side effects [99,138,140]. Lastly, we firmly believe that biological environments are required to explore and elucidate not only NPs toxicity, but also the interaction of this novel strategy both with the immune cell system, in order to avoid undesired effects, and with the serum proteins, whose binding to the NPs surfaces can significantly affect the NPs cellular uptake [141].

In general, although many challenges remain, we hypothesize that anti-glycan-mediated targeting may be a potential and valuable approach to identify the glycans on tumor cells, in which the inherent properties of the nanoparticle itself and the biological actions of anti-glycan molecules would be exploited to target tumor cells with increased efficacy and decreased non-specific effects. Certainly, the concomitant improvement in lectin engineering and mAbs production, in combination with the discovery of new GBPs, may have a great value for the implementation of the anti-glycan NPs in the cancer field.

## 7. Conclusions and Future Outlook

Nanotechnology is an emerging technology that may change the face of cancer diagnosis and therapy by the development of a new targeted system, with numerous advantages for tumor visualization and eradication. Cancer-associated glycans represent valuable opportunities for cancer diagnosis, prognosis, and therapy. As described here, the molecules showing exceptional glycan recognition property may represent a powerful strategy for the identification of altered glycans for cancer therapy and diagnosis, even if their clinical application remains limited due to concerns with stability and toxicity. We can predict that the conjugation to nanoparticles systems will hasten the translation towards the biomedical application of anti-glycan molecules. Moreover, the literature findings clearly revealed that their conjugation on the NPs could greatly increase the NPs delivery, thus minimizing/avoiding side-effects and facilitating uptake in the target cells. It is clear, however, that fine-tuning of the conjugation method of these molecules on NPs is still required in order to maintain their immunoreactivity for tumor-associated carbohydrates antigens and also to ensure the stability of the functionalized nanosystems. In conclusion, thus far there are no approved anti-glycan NPs, and, in this review, we have aimed to highlight the huge solution offered by the anti-glycan conjugated nanoparticles to greatly increase the efficacy and specificity of nanoparticles towards aberrant glycans. There is no doubt that gaining new insights on molecules that have the capacity to recognize aberrant cancer-associated glycosylation, as well as the identification of cancer-specific glycans and the optimization of nanoparticle synthesis before conjugation, will continue to be a high priority for the foreseeable future in the fight against cancer.

## Figures and Tables

**Table 1:** Nanoparticles (NPs) functionalized with lectin targeting aberrant cancer-associated glycans. Abbreviations: Jacalin of *Artocarpus integrifolia* (JCA); *Ulex europaeus* Agglutinin-1 (UEA1); Cratylia mollis seeds lectin (Cramoll); *Aleuria aurantia* lectin (ALL); *Ricinus communis* agglutinin (RCA); *Lotus tetragonolobus* lectin (LTL); 5-fluorouracil (5-FU); (−)-epigallocatechin-3-gallate (EGCG); quantum dots (QDs); polyethylene glycol (PEG); gold nanoparticles (AuNPs).

Lectin	Target	Cancer Type	Nanoparticles	Application	Reference
ConA	TF antigen	Bone cancer	DOX-loaded MSNs	Targeting Therapy	[43]
WGA	Neu5Ac GlcNac	Colon adenocarcinoma	5-FU-EGCG -gelatin-chitosan NPs	Targeting Therapy	[44]
UEA I	α-L-fucose	Colorectal cancer	Fluorescent MSNs	Targeting Diagnosis	[45]
ConA	TF antigen	Adenocarcinoma of the cervix	DOX-loaded graphene QDs -Fe_3_O_4_	Targeting Therapy Diagnosis	[46]
JCA	TF antigen	Colorectal and breast adenocarcinoma	Phthalocyanine- PEG-AuNPs	Targeting Therapy	[47]
ConA; RCA; WGA	TF antigen; β-D-galactose; Neu5Ac and GlcNac	Colorectal cancer	Fe_2_O_3_ -AuNPs	Targeting Diagnosis	[48]
Cramoll	glucose/mannose	Fibroadenoma and invasive ductal carcinoma human breast tissue	QDs	Targeting Diagnosis	[49]
ALL	Le^X^	Colon cancer	ATTO 430LS dye-loaded MSNs	Targeting Diagnosis Therapy	[50]
LTL	α-1,2-linked fucose	Prostate carcinoma and melanoma	DOX-loaded liposomes	Targeting Therapy	[51]

**Table 2:** Nanoparticles functionalized with monoclonal antibodies (mAbs) targeting aberrant cancer-associated glycans. Abbreviations: poly lactic-co-glycolic acid (PLGA); gadolinium (Gd); gemcitabine (GEM); prussian blue nanoparticles (PBNPs); sorafenib (SFB); glypican-3 (GPC-3).

Antibody	Target	Cancer Type	Nanoparticles	Application	Reference
hu14.18K322A mAb	GD2	neuroblastoma	iron-oxide NPs	Targeting Diagnosis	[71]
mAb 3F8	GD2	neuroblastoma	SN-38 loaded polymeric NPs	Targeting Therapy	[72]
ch14.18/CHO	GD2	glioblastoma	PLGA nanoparticles	Targeting Therapy	[77]
hu14.18K322A	GD2	neuroblastoma and melanoma cancers	AuNPs	Targeting Diagnosis Therapy	[78]
GPC-1 mAb	GPC-1	pancreatic cancer	Gd-Au nanoclusters	Targeting Diagnosis	[76]
GPC-1 mAb	GPC-1	pancreatic cancer	GEM-loaded multifunctional Au nanocarrier	Targeting Diagnosis Therapy	[79]
GPC-3 mAb	GPC-3	hepatocellular carcinoma	PBNPs	Targeting Diagnosis Therapy	[80]
GPC-3 mAb	GPC-3	hepatocellular carcinoma	SFB-loaded polymeric NPs	Targeting Therapy	[81]

**Table 3:** Examples of recent innovative anti-glycan approaches to target NPs to cancer cells.

Targeting Moiety	Target	Cancer Type	NPs	Application	Reference
Apt	MUC1	breast cancer	chitosan NPs	Targeting Therapy	[87]
Heptapeptide	MUC5AC	gastric cancer	USPIOs	Targeting Diagnosis	[90]
Phenylboronic Acid	sialic acid	neuroblastoma	chitosan NPs	Targeting Therapy	[93]
Monosaccharide-imprinted polymer	sialic acid, fucose or mannose	hepatoma carcinoma and breast cancer	fluorescent silica NPs	Targeting Diagnosis	[95]
HA	CD44	breast cancer	HA nanocarrier	Targeting Therapy	[106]

## References

[1] Salvioni L., Rizzuto M.A., Bertolini J.A., Pandolfi L., Colombo M., Prosperi D. (2019). Thirty Years of Cancer Nanomedicine: Success, Frustration, and Hope. Cancers.

[2] Ding Z., Sigdel K., Yang L., Liu Y., Xuan M., Wang X., Gu Z., Wu J., Xie H. (2020). Nanotechnology-Based Drug Delivery Systems for Enhanced Diagnosis and Therapy of Oral Cancer. J. Mater. Chem. B.

[3] Patra J.K., Das G., Fraceto L.F., Campos E.V.R., del Rodriguez-Torres M.P., Acosta-Torres L.S., Diaz-Torres L.A., Grillo R., Swamy M.K., Sharma S. (2018). Nano Based Drug Delivery Systems: Recent Developments and Future Prospects. J. Nanobiotechnol..

[4] Liu R., Kay B.K., Jiang S., Chen S. (2009). Nanoparticle Delivery: Targeting and Nonspecific Binding. MRS Bull..

[5] Bazak R., Houri M., Achy S.E., Hussein W., Refaat T. (2014). Passive Targeting of Nanoparticles to Cancer: A Comprehensive Review of the Literature. Mol. Clin. Oncol..

[6] Pearce A.K., O’Reilly R.K. (2019). Insights into Active Targeting of Nanoparticles in Drug Delivery: Advances in Clinical Studies and Design Considerations for Cancer Nanomedicine. Bioconj. Chem..

[7] Jahan S.T., Sadat S.M.A., Walliser M., Haddadi A. (2017). Targeted Therapeutic Nanoparticles: An Immense Promise to Fight against Cancer. J. Drug. Deliv..

[8] Allen T.M. (2002). Ligand-Targeted Therapeutics in Anticancer Therapy. Nat. Rev. Cancer.

[9] Yoo J., Park C., Yi G., Lee D., Koo H. (2019). Active Targeting Strategies Using Biological Ligands for Nanoparticle Drug Delivery Systems. Cancers.

[10] Bloise N., Massironi A., Pina C.D., Alongi J., Siciliani S., Manfredi A., Biggiogera M., Rossi M., Ferruti P., Ranucci E. (2020). Extra-Small Gold Nanospheres Decorated with a Thiol Functionalized Biodegradable and Biocompatible Linear Polyamidoamine as Nanovectors of Anticancer Molecules. Front. Bioeng. Biotechnol..

[11] Friedman A.D., Claypool S.E., Liu R. (2013). The Smart Targeting of Nanoparticles. Curr. Pharm. Des..

[12] Clift M.J.D., Rothen-Rutishauser B., Brown D.M., Duffin R., Donaldson K., Proudfoot L., Guy K., Stone V. (2008). The Impact of Different Nanoparticle Surface Chemistry and Size on Uptake and Toxicity in a Murine Macrophage Cell Line. Toxicol. Appl. Pharmacol..

[13] Narkhede A.A., Sherwood J.A., Antone A., Coogan K.R., Bolding M.S., Deb S., Bao Y., Rao S.S. (2019). Role of Surface Chemistry in Mediating the Uptake of Ultrasmall Iron Oxide Nanoparticles by Cancer Cells. ACS Appl. Mater. Interfaces.

[14] Sherwood J., Lovas K., Rich M., Yin Q., Lackey K., Bolding M.S., Bao Y. (2016). Shape-Dependent Cellular Behaviors and Relaxivity of Iron Oxide-Based T1 MRI Contrast Agents. Nanoscale.

[15] Nag S., Manna K., Saha K.D. (2019). Tannic Acid-Stabilized Gold Nano-Particles Are Superior to Native Tannic Acid in Inducing ROS-Dependent Mitochondrial Apoptosis in Colorectal Carcinoma Cells via the P53/AKT Axis. RSC Adv..

[16] Shin M., Ryu J.H., Park J., Kim K., Yang J., Lee H. (2015). DNA/Tannic Acid Hybrid Gel Exhibiting Biodegradability, Extensibility, Tissue Adhesiveness, and Hemostatic Ability. Adv. Funct. Mater..

[17] Sahiner N., Sagbas S., Aktas N. (2015). Single Step Natural Poly(Tannic Acid) Particle Preparation as Multitalented Biomaterial. Mater. Sci. Eng. C Mater. Biol. Appl..

[18] Aguilera J.R., Venegas V., Oliva J.M., Sayagués M.J., de Miguel M., Sánchez-Alcázar J.A., Arévalo-Rodríguez M., Zaderenko A.P. (2016). Targeted Multifunctional Tannic Acid Nanoparticles. RSC Adv..

[19] Amoozgar Z., Park J., Lin Q., Weidle J.H., Yeo Y. (2013). Development of Quinic Acid-Conjugated Nanoparticles as a Drug Carrier to Solid Tumors. Biomacromolecules.

[20] Varki A. (2017). Biological Roles of Glycans. Glycobiology.

[21] Liu H.-M., Ma L., Cao B., Lin J.-Z., Han L., Li C.-Y., Xu R.-C., Zhang D.-K. (2020). Progress in Research into the Role of Abnormal Glycosylation Modification in Tumor Immunity. Immunol. Lett..

[22] Ohtsubo K., Marth J.D. (2006). Glycosylation in Cellular Mechanisms of Health and Disease. Cell.

[23] Gupta R., Leon F., Rauth S., Batra S.K., Ponnusamy M.P. (2020). A Systematic Review on the Implications of O-Linked Glycan Branching and Truncating Enzymes on Cancer Progression and Metastasis. Cells.

[24] Wang M., Zhu J., Lubman D.M., Gao C. (2019). Aberrant Glycosylation and Cancer Biomarker Discovery: A Promising and Thorny Journey. Clin. Chem. Lab. Med..

[25] Inagaki Y., Song P., Tang W., Kokudo N. (2015). Cancer-Associated Carbohydrate Antigens for Clinical Diagnostic Markers—Its Effectiveness and Limitations. Drug Discov. Ther..

[26] Costa A.F., Campos D., Reis C.A., Gomes C. (2020). Targeting Glycosylation: A New Road for Cancer Drug Discovery. Trends Cancer.

[27] Mereiter S., Balmaña M., Campos D., Gomes J., Reis C.A. (2019). Glycosylation in the Era of Cancer-Targeted Therapy: Where Are We Heading?. Cancer Cell.

[28] Thomas D., Rathinavel A.K., Radhakrishnan P. (2020). Altered Glycosylation in Cancer: A Promising Target for Biomarkers and Therapeutics. Biochim. Biophys. Acta Rev. Cancer.

[29] Padler-Karavani V. (2014). Aiming at the Sweet Side of Cancer: Aberrant Glycosylation as Possible Target for Personalized-Medicine. Cancer Lett..

[30] Fernandes E., Sores J., Cotton S., Peixoto A., Ferreira D., Freitas R., Reis C.A., Santos L.L., Ferreira J.A. (2020). Esophageal, Gastric and Colorectal Cancers: Looking beyond Classical Serological Biomarkers towards Glycoproteomics-Assisted Precision Oncology. Theranostics.

[31] Arnaud J., Audfray A., Imberty A. (2013). Binding Sugars: From Natural Lectins to Synthetic Receptors and Engineered Neolectins. Chem. Soc. Rev..

[32] Tommasone S., Allabush F., Tagger Y.K., Norman J., Köpf M., Tucker J.H.R., Mendes P.M. (2019). The Challenges of Glycan Recognition with Natural and Artificial Receptors. Chem. Soc. Rev..

[33] Bhutia S.K., Panda P.K., Sinha N., Praharaj P.P., Bhol C.S., Panigrahi D.P., Mahapatra K.K., Saha S., Patra S., Mishra S.R. (2019). Plant Lectins in Cancer Therapeutics: Targeting Apoptosis and Autophagy-Dependent Cell Death. Pharmacol. Res..

[34] Coulibaly F.S., Youan B.-B.C. (2017). Current Status of Lectin-Based Cancer Diagnosis and Therapy. AIMS Mol. Sci..

[35] Poiroux G., Barre A., Van Damme E.J.M., Benoist H., Rougé P. (2017). Plant Lectins Targeting O-Glycans at the Cell Surface as Tools for Cancer Diagnosis, Prognosis and Therapy. Int. J. Mol. Sci..

[36] Bovi M., Cenci L., Perduca M., Capaldi S., Carrizo M.E., Civiero L., Chiarelli L.R., Galliano M., Monaco H.L. (2013). BEL β-trefoil: A novel lectin with antineoplastic properties in king bolete (Boletus edulis) mushrooms. Glycobiology.

[37] Yau T., Dan X., Ng C.C.W., Ng T.B. (2015). Lectins with Potential for Anti-Cancer Therapy. Molecules.

[38] Chang C.-P., Yang M.-C., Liu H.-S., Lin Y.-S., Lei H.-Y. (2007). Concanavalin A Induces Autophagy in Hepatoma Cells and Has a Therapeutic Effect in a Murine in Situ Hepatoma Model. Hepatology.

[39] Perduca M., Carbonare L.D., Bovi M., Innamorati G., Cheri S., Cavallini C., Scupoli M.T., Mori A., Valenti M.T. (2017). Runx2 Downregulation, Migration and Proliferation Inhibition in Melanoma Cells Treated with BEL β-Trefoil. Oncol. Rep..

[40] Souza M.A., Carvalho F.C., Ruas L.P., Ricci-Azevedo R., Roque-Barreira M.C. (2013). The Immunomodulatory Effect of Plant Lectins: A Review with Emphasis on ArtinM Properties. Glycoconj. J..

[41] Ernst E., Schmidt K., Steuer-Vogt M.K. (2003). Mistletoe for Cancer? A Systematic Review of Randomised Clinical Trials. Int. J. Cancer.

[42] He S., Simpson B.K., Sun H., Ngadi M.O., Ma Y., Huang T. (2018). Phaseolus Vulgaris Lectins: A Systematic Review of Characteristics and Health Implications. Crit. Rev. Food Sci. Nutr..

[43] Martínez-Carmona M., Lozano D., Colilla M., Vallet-Regí M. (2018). Lectin-Conjugated PH-Responsive Mesoporous Silica Nanoparticles for Targeted Bone Cancer Treatment. Acta Biomater..

[44] Wang R., Huang J., Chen J., Yang M., Wang H., Qiao H., Chen Z., Hu L., Di L., Li J. (2019). Enhanced Anti-Colon Cancer Efficacy of 5-Fluorouracil by Epigallocatechin-3- Gallate Co-Loaded in Wheat Germ Agglutinin-Conjugated Nanoparticles. Nanomedicine.

[45] Chen N.-T., Souris J.S., Cheng S.-H., Chu C.-H., Wang Y.-C., Konda V., Dougherty U., Bissonnette M., Mou C.-Y., Chen C.-T. (2017). Lectin-Functionalized Mesoporous Silica Nanoparticles for Endoscopic Detection of Premalignant Colonic Lesions. Nanomedicine.

[46] Chowdhury A.D., Ganganboina A.B., Tsai Y., Chiu H., Doong R. (2018). Multifunctional GQDs-Concanavalin A@Fe3O4 Nanocomposites for Cancer Cells Detection and Targeted Drug Delivery. Anal. Chim. Acta.

[47] Obaid G., Chambrier I., Cook M.J., Russell D.A. (2015). Cancer Targeting with Biomolecules: A Comparative Study of Photodynamic Therapy Efficacy Using Antibody or Lectin Conjugated Phthalocyanine-PEG Gold Nanoparticles. Photochem. Photobiol. Sci..

[48] He X., Liu F., Liu L., Duan T., Zhang H., Wang Z. (2014). Lectin-Conjugated Fe_2_O_3_@Au Core@Shell Nanoparticles as Dual Mode Contrast Agents for in Vivo Detection of Tumor. Mol. Pharm..

[49] Carvalho M.E.T., Oliveira W.F., Cunha C.R.A., Coelho L.C.B.B., Silva M.V., Junior L.B.C., Santos B.S., Filho P.E.C., Fontes A., Correia M.T.S. (2019). Evaluating the Glycophenotype on Breast Cancer Tissues with Quantum Dots-Cramoll Lectin Conjugates. Int. J. Biol. Macromol..

[50] Bhat R., García I., Aznar E., Arnaiz B., Martínez-Bisbal M.C., Liz-Marzán L.M., Penadés S., Martínez-Máñez R. (2017). Lectin-Gated and Glycan Functionalized Mesoporous Silica Nanocontainers for Targeting Cancer Cells Overexpressing Lewis X Antigen. Nanoscale.

[51] Giovampaola C.D., Capone A., Ermini L., Lupetti P., Vannuccini E., Finetti F., Donnini S., Ziche M., Magnani A., Leone G. (2017). Formulation of Liposomes Functionalized with Lotus Lectin and Effective in Targeting Highly Proliferative Cells. Biochim. Biophys. Acta Gen. Subj..

[52] Soliman C., Yuriev E., Ramsland P.A. (2017). Antibody Recognition of Aberrant Glycosylation on the Surface of Cancer Cells. Curr. Opin. Struct. Biol..

[53] Sterner E., Flanagan N., Gildersleeve J.C. (2016). Perspectives on Anti-Glycan Antibodies Gleaned from Development of a Community Resource Database. ACS Chem. Biol..

[54] Redman J.M., Hill E.M., Al Deghaither D., Weiner L.M. (2015). Mechanisms of Action of Therapeutic Antibodies for Cancer. Mol. Immunol..

[55] Chua J.X., Durrant L. (2017). Monoclonal Antibodies against Tumour-Associated Carbohydrate Antigens. Carbohydrate.

[56] Mantuano N.R., Natoli M., Zippelius A., Läubli H. (2020). Tumor-Associated Carbohydrates and Immunomodulatory Lectins as Targets for Cancer Immunotherapy. J. Immunother. Cancer.

[57] Keyel M.E., Reynolds C.P. (2019). Spotlight on Dinutuximab in the Treatment of High-Risk Neuroblastoma: Development and Place in Therapy. Biologics.

[58] Suzuki M., Cheung N.-K.V. (2015). Disialoganglioside GD2 as a Therapeutic Target for Human Diseases. Expert Opin. Ther. Targets.

[59] Tolcher A.W., Sugarman S., Gelmon K.A., Cohen R., Saleh M., Isaacs C., Young L., Healey D., Onetto N., Slichenmyer W. (1999). Randomized Phase II Study of BR96-Doxorubicin Conjugate in Patients with Metastatic Breast Cancer. J. Clin. Oncol..

[60] Scott A.M., Geleick D., Rubira M., Clarke K., Nice E.C., Smyth F.E., Stockert E., Richards E.C., Carr F.J., Harris W.J. (2000). Construction, Production, and Characterization of Humanized Anti-Lewis Y Monoclonal Antibody 3S193 for Targeted Immunotherapy of Solid Tumors. Cancer Res..

[61] Farrugia W., Scott A.M., Ramsland P.A. (2009). A Possible Role for Metallic Ions in the Carbohydrate Cluster Recognition Displayed by a Lewis Y Specific Antibody. PLoS ONE.

[62] Amon R., Rosenfeld R., Perlmutter S., Grant O.C., Yehuda S., Borenstein-Katz A., Alcalay R., Marshanski T., Yu H., Diskin R. (2020). Directed Evolution of Therapeutic Antibodies Targeting Glycosylation in Cancer. Cancers.

[63] Kiessling L.L., Grim J.C. (2013). Glycopolymer Probes of Signal Transduction. Chem. Soc. Rev..

[64] Bashir S., Arye S.L.B., Reuven E.M., Yu H., Costa C., Galiñanes M., Bottio T., Chen X., Padler-Karavani V. (2019). Presentation Mode of Glycans Affect Recognition of Human Serum Anti-Neu5Gc IgG Antibodies. Bioconj. Chem..

[65] Marques A.C., Costa P.J., Velho S., Amaral M.H. (2020). Functionalizing Nanoparticles with Cancer-Targeting Antibodies: A Comparison of Strategies. J. Control. Release.

[66] Qiu H., Min Y., Rodgers Z., Zhang L., Wang A.Z. (2017). Nanomedicine Approaches to Improve Cancer Immunotherapy. Wiley Interdiscip. Rev. Nanomed. Nanobiotechnol..

[67] Gao X., Li L., Cai X., Huang Q., Xiao J., Cheng Y. (2021). Targeting Nanoparticles for Diagnosis and Therapy of Bone Tumors: Opportunities and Challenges. Biomaterials.

[68] Arruebo M., Valladares M., González-Fernández Á. (2009). Antibody-Conjugated Nanoparticles for Biomedical Applications. J. Nanomater..

[69] Rodríguez-Nogales C., Noguera R., Couvreur P., Blanco-Prieto M.J. (2019). Therapeutic Opportunities in Neuroblastoma Using Nanotechnology. J. Pharmacol. Exp. Ther..

[70] Gholizadeh S., Dolman E.M., Wieriks R., Sparidans R.W., Hennink W.E., Kok R.J. (2018). Anti-GD2 Immunoliposomes for Targeted Delivery of the Survivin Inhibitor Sepantronium Bromide (YM155) to Neuroblastoma Tumor Cells. Pharm. Res..

[71] Baiu D.C., Artz N.S., McElreath M.R., Menapace B.D., Hernando D., Reeder S.B., Grüttner C., Otto M. (2015). High Specificity Targeting and Detection of Human Neuroblastoma Using Multifunctional Anti-GD2 Iron-Oxide Nanoparticles. Nanomedicine.

[72] Monterrubio C., Paco S., Olaciregui N.G., Pascual-Pasto G., Vila-Ubach M., Cuadrado-Vilanova M., Ferrandiz M.M., Castillo-Ecija H., Glisoni R., Kuplennik N. (2017). Targeted Drug Distribution in Tumor Extracellular Fluid of GD2-Expressing Neuroblastoma Patient-Derived Xenografts Using SN-38-Loaded Nanoparticles Conjugated to the Monoclonal Antibody 3F8. J. Control. Release.

[73] Nazha B., Inal C., Owonikoko T.K. (2020). Disialoganglioside GD2 Expression in Solid Tumors and Role as a Target for Cancer Therapy. Front. Oncol..

[74] Melo S.A., Luecke L.B., Kahlert C., Fernandez A.F., Gammon S.T., Kaye J., LeBleu V.S., Mittendorf E.A., Weitz J., Rahbari N. (2015). Glypican-1 Identifies Cancer Exosomes and Detects Early Pancreatic Cancer. Nature.

[75] Kleeff J., Ishiwata T., Kumbasar A., Friess H., Büchler M.W., Lander A.D., Korc M. (1998). The Cell-Surface Heparan Sulfate Proteoglycan Glypican-1 Regulates Growth Factor Action in Pancreatic Carcinoma Cells and Is Overexpressed in Human Pancreatic Cancer. J. Clin. Investig..

[76] Huang X., Fan C., Zhu H., Le W., Cui S., Chen X., Li W., Zhang F., Huang Y., Shi D. (2018). Glypican-1-Antibody-Conjugated Gd-Au Nanoclusters for FI/MRI Dual-Modal Targeted Detection of Pancreatic Cancer. IJN.

[77] Tivnan A., Heilinger T., Ramsey J.M., O’Connor G., Pokorny J.L., Sarkaria J.N., Stringer B.W., Day B.W., Boyd A.W., Kim E.L. (2017). Anti-GD2-Ch14.18/CHO Coated Nanoparticles Mediate Glioblastoma (GBM)-Specific Delivery of the Aromatase Inhibitor, Letrozole, Reducing Proliferation, Migration and Chemoresistance in Patient-Derived GBM Tumor Cells. Oncotarget.

[78] Jiao P., Otto M., Geng Q., Li C., Li F., Butch E.R., Snyder S.E., Zhou H., Yan B. (2016). Enhancing Both CT Imaging and Natural Killer Cell-Mediated Cancer Cell Killing by a GD2-Targeting Nanoconstruct. J. Mater. Chem. B.

[79] Qiu W., Zhang H., Chen X., Song L., Cui W., Ren S., Wang Y., Guo K., Li D., Chen R. (2019). A GPC1-Targeted and Gemcitabine-Loaded Biocompatible Nanoplatform for Pancreatic Cancer Multimodal Imaging and Therapy. Nanomedicine.

[80] Li Z., Zeng Y., Zhang D., Wu M., Wu L., Huang A., Yang H., Liu X., Liu J. (2014). Glypican-3 Antibody Functionalized Prussian Blue Nanoparticles for Targeted MR Imaging and Photothermal Therapy of Hepatocellular Carcinoma. J. Mater. Chem. B.

[81] Tang X., Chen L., Li A., Cai S., Zhang Y., Liu X., Jiang Z., Liu X., Liang Y., Ma D. (2018). Anti-GPC3 Antibody-Modified Sorafenib-Loaded Nanoparticles Significantly Inhibited HepG2 Hepatocellular Carcinoma. Drug Deliv..

[82] Li W., Ma Y., Guo Z., Xing R., Liu Z. (2020). Efficient Screening of Glycan-Specific Aptamers Using a Glycosylated Peptide as a Scaffold. Anal. Chem..

[83] Nabavinia M.S., Gholoobi A., Charbgoo F., Nabavinia M., Ramezani M., Abnous K. (2017). Anti-MUC1 Aptamer: A Potential Opportunity for Cancer Treatment. Med. Res. Rev..

[84] Nath S., Mukherjee P. (2014). Muc1: A Multifaceted Oncoprotein with a Key Role in Cancer Progression. Trends Mol. Med..

[85] Ferreira C.S.M., Matthews C.S., Missailidis S. (2006). DNA Aptamers That Bind to MUC1 Tumour Marker: Design and Characterization of MUC1-Binding Single-Stranded DNA Aptamers. Tumour. Biol..

[86] Sayari E., Dinarvand M., Amini M., Azhdarzadeh M., Mollarazi E., Ghasemi Z., Atyabi F. (2014). MUC1 Aptamer Conjugated to Chitosan Nanoparticles, an Efficient Targeted Carrier Designed for Anticancer SN38 Delivery. Int. J. Pharm..

[87] Jafari R., Zolbanin N.M., Majidi J., Atyabi F., Yousefi M., Jadidi-Niaragh F., Aghebati-Maleki L., Shanehbandi D., Zangbar M.-S.S., Rafatpanah H. (2019). Anti-Mucin1 Aptamer-Conjugated Chitosan Nanoparticles for Targeted Co-Delivery of Docetaxel and IGF-1R SiRNA to SKBR3 Metastatic Breast Cancer Cells. Iran. Biomed. J..

[88] Houvast R.D., Vankemmelbeke M., Durrant L.G., Wuhrer M., Baart V.M., Kuppen P.J.K., de Geus-Oei L.-F., Vahrmeijer A.L., Sier C.F.M. (2020). Targeting Glycans and Heavily Glycosylated Proteins for Tumor Imaging. Cancers.

[89] Zhao H., Richardson R., Talebloo N., Mukherjee P., Wang P., Moore A. (2019). UMUC1-Targeting Magnetic Resonance Imaging of Therapeutic Response in an Orthotropic Mouse Model of Colon Cancer. Mol. Imaging Biol..

[90] Rossez Y., Burtea C., Laurent S., Gosset P., Léonard R., Gonzalez W., Ballet S., Raynal I., Rousseaux O., Dugué T. (2016). Early Detection of Colonic Dysplasia by Magnetic Resonance Molecular Imaging with a Contrast Agent Raised against the Colon Cancer Marker MUC5AC. Contrast Media Mol. Imaging.

[91] Yang W., Fan H., Gao X., Gao S., Karnati V.V.R., Ni W., Hooks W.B., Carson J., Weston B., Wang B. (2004). The First Fluorescent Diboronic Acid Sensor Specific for Hepatocellular Carcinoma Cells Expressing Sialyl Lewis X. Chem. Biol..

[92] Qian X., Ge L., Yuan K., Li C., Zhen X., Cai W., Cheng R., Jiang X. (2019). Targeting and Microenvironment-Improving of Phenylboronic Acid-Decorated Soy Protein Nanoparticles with Different Sizes to Tumor. Theranostics.

[93] Wang X., Tang H., Wang C., Zhang J., Wu W., Jiang X. (2016). Phenylboronic Acid-Mediated Tumor Targeting of Chitosan Nanoparticles. Theranostics.

[94] Yin D., Li X., Ma Y., Liu Z. (2017). Targeted Cancer Imaging and Photothermal Therapy via Monosaccharide-Imprinted Gold Nanorods. Chem. Commun..

[95] Wang S., Yin D., Wang W., Shen X., Zhu J.-J., Chen H.-Y., Liu Z. (2016). Targeting and Imaging of Cancer Cells via Monosaccharide-Imprinted Fluorescent Nanoparticles. Sci. Rep..

[96] Vlatakis G., Andersson L.I., Müller R., Mosbach K. (1993). Drug Assay Using Antibody Mimics Made by Molecular Imprinting. Nature.

[97] Fernandes E., Ferreira D., Peixoto A., Freitas R., Relvas-Santos M., Palmeira C., Martins G., Barros A., Santos L.L., Sarmento B. (2019). Glycoengineered Nanoparticles Enhance the Delivery of 5-Fluoroucil and Paclitaxel to Gastric Cancer Cells of High Metastatic Potential. Int. J. Pharm..

[98] Choi K.Y., Yoon H.Y., Kim J.-H., Bae S.M., Park R.-W., Kang Y.M., Kim I.-S., Kwon I.C., Choi K., Jeong S.Y. (2011). Smart Nanocarrier Based on PEGylated Hyaluronic Acid for Cancer Therapy. ACS Nano.

[99] Sampaolesi S., Nicotra F., Russo L. (2018). Glycans in Nanomedicine, Impact and Perspectives. Future Med. Chem..

[100] Turcsányi Á., Varga N., Csapó E. (2020). Chitosan-modified hyaluronic acid-based nanosized drug carriers. Int J Biol Macromol..

[101] Kim J.H., Moon M.J., Kim D.Y., Heo S.H., Jeong Y.Y. (2018). Hyaluronic Acid-Based Nanomaterials for Cancer Therapy. Polymers.

[102] Azevedo R., Gaiteiro C., Peixoto A., Relvas-Santos M., Lima L., Santos L.L., Ferreira J.A. (2018). CD44 Glycoprotein in Cancer: A Molecular Conundrum Hampering Clinical Applications. Clin. Proteom..

[103] Lepenies B., Lee J., Sonkaria S. (2013). Targeting C-Type Lectin Receptors with Multivalent Carbohydrate Ligands. Adv. Drug Deliv. Rev..

[104] Hatami E., Mu Y., Shields D.N., Chauhan S.C., Kumar S., Cory T.J., Yallapu M.M. (2019). Mannose-Decorated Hybrid Nanoparticles for Enhanced Macrophage Targeting. Biochem. Biophys. Rep..

[105] Lemarchand C., Gref R., Couvreur P. (2004). Polysaccharide-Decorated Nanoparticles. Eur. J. Pharm. Biopharm..

[106] Yu T., Li Y., Gu X., Li Q. (2020). Development of a Hyaluronic Acid-Based Nanocarrier Incorporating Doxorubicin and Cisplatin as a PH-Sensitive and CD44-Targeted Anti-Breast Cancer Drug Delivery System. Front. Pharmacol..

[107] Yang M., Li J., Gu P., Fan X. (2021). The Application of Nanoparticles in Cancer Immunotherapy: Targeting Tumor Microenvironment. Bioact. Mater..

[108] Korangath P., Barnett J.D., Sharma A., Henderson E.T., Stewart J., Yu S.-H., Kandala S.K., Yang C.-T., Caserto J.S., Hedayati M. (2020). Nanoparticle Interactions with Immune Cells Dominate Tumor Retention and Induce T Cell–Mediated Tumor Suppression in Models of Breast Cancer. Sci. Adv..

[109] Baeza A. (2020). Tumor Targeted Nanocarriers for Immunotherapy. Molecules.

[110] Du J., Zhang Y.S., Hobson D., Hydbring P. (2017). Nanoparticles for Immune System Targeting. Drug Discov. Today.

[111] Irvine D.J., Dane E.L. (2020). Enhancing Cancer Immunotherapy with Nanomedicine. Nat. Rev. Immunol..

[112] Whiteside T. (2008). The Tumor Microenvironment and Its Role in Promoting Tumor Growth. Oncogene.

[113] Mantuano N.R., Oliveira-Nunes M.C., Alisson-Silva F., Dias W.B., Todeschini A.R. (2019). Emerging Role of Glycosylation in the Polarization of Tumor-Associated Macrophages. Pharmacol. Res..

[114] Moriwaki K., Asahi M. (2017). Augmented TME O-GlcNAcylation Promotes Tumor Proliferation through the Inhibition of P38 MAPK. Mol. Cancer Res..

[115] Kilpatrick D.C. (2002). Animal Lectins: A Historical Introduction and Overview. Biochim. Biophys. Acta Gen. Subj..

[116] Busold S., Nagy N.A., Tas S.W., van Ree R., de Jong E.C., Geijtenbeek T.B.H. (2020). Various Tastes of Sugar: The Potential of Glycosylation in Targeting and Modulating Human Immunity via C-Type Lectin Receptors. Front. Immunol..

[117] Cornelissen L.A.M., Van Vliet S.J. (2016). A Bitter Sweet Symphony: Immune Responses to Altered O-Glycan Epitopes in Cancer. Biomolecules.

[118] Streng-Ouwehand I., Unger W.W.J., Van Kooyk Y. (2011). C-Type Lectin Receptors for Tumor Eradication: Future Directions. Cancers.

[119] Hockl P.F., Wolosiuk A., Pérez-Sáez J.M., Bordoni A.V., Croci D.O., Toum-Terrones Y., Soler-Illia G.J.A.A., Rabinovich G.A. (2016). Glyco-Nano-Oncology: Novel Therapeutic Opportunities by Combining Small and Sweet. Pharmacol. Res..

[120] Solinas G., Germano G., Mantovani A., Allavena P. (2009). Tumor-Associated Macrophages (TAM) as Major Players of the Cancer-Related Inflammation. J. Leukoc. Biol..

[121] Su Y., Bakker T., Harris J., Tsang C., Brown G.D., Wormald M.R., Gordon S., Dwek R.A., Rudd P.M., Martinez-Pomares L. (2005). Glycosylation Influences the Lectin Activities of the Macrophage Mannose Receptor. J. Biol. Chem..

[122] Stanczak M.A., Siddiqui S.S., Trefny M.P., Thommen D.S., Boligan K.F., von Gunten S., Tzankov A., Tietze L., Lardinois D., Heinzelmann-Schwarz V. (2018). Self-associated molecular patterns mediate cancer immune evasion by engaging Siglecs on T cells. J Clin Investig..

[123] Galectin-3 Is a Negative Regulator of Lipopolysaccharide-Mediated Inflammation|the Journal of Immunology. https://www.jimmunol.org/content/181/4/2781.

[124] Ovais M., Guo M., Chen C. (2019). Tailoring Nanomaterials for Targeting Tumor-Associated Macrophages. Adv. Mater..

[125] Altevogt P., Sammar M., Hüser L., Kristiansen G. (2021). Novel Insights into the Function of CD24: A Driving Force in Cancer. Int. J. Cancer.

[126] Ayre D.C., Pallegar N.K., Fairbridge N.A., Canuti M., Lang A.S., Christian S.L. (2016). Analysis of the Structure, Evolution, and Expression of CD24, an Important Regulator of Cell Fate. Gene.

[127] Polli C.D., Ruas L.P., Veronez L.C., Geraldino T.H., de Morais F.R., Roque-Barreira M.C., da-Silva G.P. (2016). Jacalin-Activated Macrophages Exhibit an Antitumor Phenotype. Biomed. Res. Int..

[128] Bhutia S.K., Mallick S.K., Maiti T.K. (2009). In Vitro Immunostimulatory Properties of Abrus Lectins Derived Peptides in Tumor Bearing Mice. Phytomedicine.

[129] Heimburg-Molinaro J., Lum M., Vijay G., Jain M., Almogren A., Rittenhouse-Olson K. (2011). Cancer Vaccines and Carbohydrate Epitopes. Vaccine.

[130] Polonskaya Z., Savage P.B., Finn M.G., Teyton L. (2019). High-Affinity Anti-Glycan Antibodies: Challenges and Strategies. Curr. Opin. Immunol..

[131] Shi J., Kantoff P.W., Wooster R., Farokhzad O.C. (2017). Cancer Nanomedicine: Progress, Challenges and Opportunities. Nat. Rev. Cancer.

[132] Villarreal E., Li G.G., Zhang Q., Fu X., Wang H. (2017). Nanoscale Surface Curvature Effects on Ligand–Nanoparticle Interactions: A Plasmon-Enhanced Spectroscopic Study of Thiolated Ligand Adsorption, Desorption, and Exchange on Gold Nanoparticles. Nano Lett..

[133] Duan X., Li Y. (2013). Physicochemical Characteristics of Nanoparticles Affect Circulation, Biodistribution, Cellular Internalization, and Trafficking. Small.

[134] Guo S., Xu C., Yin H., Hill J., Pi F., Guo P. (2020). Tuning the Size, Shape and Structure of RNA Nanoparticles for Favorable Cancer Targeting and Immunostimulation. Wiley Interdiscip. Rev. Nanomed. Nanobiotechnol..

[135] Ding L., Yao C., Yin X., Li C., Huang Y., Wu M., Wang B., Guo X., Wang Y., Wu M. (2018). Size, Shape, and Protein Corona Determine Cellular Uptake and Removal Mechanisms of Gold Nanoparticles. Small.

[136] Zhang H., Wu T., Yu W., Ruan S., He Q., Gao H. (2018). Ligand Size and Conformation Affect the Behavior of Nanoparticles Coated with in Vitro and in Vivo Protein Corona. ACS Appl. Mater. Interfaces.

[137] Fam S.Y., Chee C.F., Yong C.Y., Ho K.L., Mariatulqabtiah A.R., Tan W.S. (2020). Stealth Coating of Nanoparticles in Drug-Delivery Systems. Nanomaterials.

[138] Calzoni E., Cesaretti A., Polchi A., Di Michele A., Tancini B., Emiliani C. (2019). Biocompatible Polymer Nanoparticles for Drug Delivery Applications in Cancer and Neurodegenerative Disorder Therapies. J. Funct. Biomater..

[139] Thi T.T.H., Pilkington E.H., Nguyen D.H., Lee J.S., Park K.D., Truong N.P. (2020). The Importance of Poly(Ethylene Glycol) Alternatives for Overcoming PEG Immunogenicity in Drug Delivery and Bioconjugation. Polymers.

[140] Jhaveri J., Raichura Z., Khan T., Momin M., Omri A. (2021). Chitosan Nanoparticles-Insight into Properties, Functionalization and Applications in Drug Delivery and Theranostics. Molecules.

[141] Liu N., Tang M., Ding J. (2020). The Interaction between Nanoparticles-Protein Corona Complex and Cells and Its Toxic Effect on Cells. Chemosphere.

